# The racialization of expertise and professional non-equivalence in the humanitarian workplace

**DOI:** 10.1186/s41018-021-00112-9

**Published:** 2022-01-07

**Authors:** Junru Bian

**Affiliations:** School of Political Studies, University of Ottawa, Ottawa, Ontario Canada

**Keywords:** Race, Identity, Humanitarianism, Aid, Expertise, Racialization

## Abstract

This paper aims to explore the ways which expertise is covertly racialized in the contemporary humanitarian aid sector. While there are considerable discussions on the expat-local divide among aid professionals, such dichotomization is still inherently nationality-based, which may be an over-simplified explanation of the group dimensions within aid organizations. This study seeks to uncover that professional categorizations of “expatriate” and “local” are not race-neutral and, instead, colorblind. Organizations within the contemporary humanitarian aid apparatus have come to appeal to what Michael Omi and Howard Winant would characterize as a new racial discourse—one that does not require explicit references to race in order to be perpetuated, as racial subordination has been reconfigured to rely on implicit references to race woven within the everyday social fabrics of the humanitarian profession. The research suggests that embedded under the contemporary professional structure of the liberal humanitarian space is a covert power hierarchy fueled by perceptions of expertise and competency along racial lines—particularly around one’s whiteness.

## Background

The end of the colonial era saw the emergence of a new paradigm for humanitarianism in the global South^1^. As former imperial states retreated from their occupied territories, newly independent states found themselves in dire need to establish their own institutions to govern their development. Vast institutional vacuums appeared in states of the global South, which were quickly filled by liberal, Western non-government and international organizations. They pledged to not only relieve the suffering caused by man-made conflict and natural disasters, but also help communities with moving towards modernity and development (Barnett 2005). Aid organizations have navigated through complex humanitarian crises, namely those that took place in Somalia, Yugoslavia, and Rwanda. Their experiences in the field not only challenged the international perception on the sacrosanct identity of humanitarian organizations in being able to deliver aid wherever needed but also brought realization to organizations themselves that an altruistic humanitarian ideal alone might not be a sufficient enabler for aid operationalization.

The processes of improvisation, adaptation, and learning-by-doing were not always reliable, and that more structured, coordinated, and standardized understanding of aid operationalization in unstable contexts was needed (O’Flaherty and Ulrich 2010). As a result, the international aid apparatus gradually institutionalized, and humanitarianism saw its professionalization over time (Walker et al. 2010). The humanitarian operational process has moved to a secularized era where institutionalized organizations contractually recruit, train, and deploy professionals to implement aid programs through standardized frameworks that can be tracked, monitored, and evaluated (Barnett 2011). Career positions, such as program coordinators, gender consultants, nutrition specialists, and country directors, as well as finance officers, human resources, and procurement managers, came to characterize constituents of the contemporary global humanitarian landscape (James 2016; Weiss 2013).

Positions within international aid organizations are occupied by two distinct types of professionals. There are the *expatriate staff*—deployed by humanitarian and development aid organizations to unstable states for short periods time, often from 3 months to a year, they are mostly seen in leadership, consultancy, advisory, and decision-making positions. They are well-compensated—much more than their local counterparts. They speak in technical jargons that outsiders to the humanitarian aid apparatus can rarely understand, mostly accumulated through higher education and years of experience traversing the global South during their prior deployments. Most of them have their own cliques—often inwardly socializing within their own expat bubble, traveling in “big sport utility vehicles (SUVs) marked with the logos of their organizations” (Autesserre 2014), spending their after-work hours in restaurants, cafés, and entertainment venues that mostly foreigners can afford to frequent.

The expat bubble, although invisible, is often impenetrable for*local staff*, who are nationals of the host state where aid organizations operate. They possess specific knowledge regarding the socio-political dimensions of local communities, speak local languages, and have mostly never worked for their organizations’ other programs abroad. Local staff members are found more commonly facilitating ground-level, physical aid delivery, monitoring, and information retention positions. At the same time, it is uncommon to see local nationals occupying higher, country-level leadership posts and even more rarely as chiefs of party or country directors. They are paid according to local market salary standards, meaning that often times, their compensations are significantly lower than those of their expat counterparts.

Even when no job posting description explicitly indicate that certain positions are reserved for professionals of particular racial identities, Western, white personnel occupy large numbers of higher-level advisory, leadership and consultancy expatriate positions. Meanwhile, the majority of Black, Indigenous, and People of Color (BIPOC) professionals are seen serving in positions in “the field,” within the localities where they are born and raised, following the directory and supervision of their expatriate counterparts (Shevchenko and Fox 2008). This is not a coincidence; professional categorizations of “expatriate” and “local” are not race neutral (Kothari 2006). Instead, they are colorblind—organizations within the contemporary humanitarian aid apparatus have come to appeal to what Michael Omi and Howard Winant would characterize as a new racial discourse. It does not require explicit references to race in order to be perpetuated, as racial subordination has been reconfigured to rely on implicit references to race woven within the everyday social fabrics of the humanitarian profession (Omi and Winant 2012). Adia Benton insightfully highlights that race serves as the structural underpinning of a “non-equivalence, or the unequal valuing, of lives in the humanitarian practice (Benton 2016a).” She also reasonably underlines how racial discourses are rarely explicitly featured in the verbalities of the humanitarian practice. Taking on this understanding, I aim to examine how the uneven and fragmented criteria of being considered as a professional in the humanitarian sector are contingent upon this exact under-recognized, if not under-realized, non-equivalence. In turn, this paper suggests that embedded under the contemporary professional structure of the liberal humanitarian space is a covert power hierarchy fueled by perceptions of expertise and competency along racial lines—particularly around one’s whiteness.

## Racial formation and colorblindness

For the purpose of the research, I reference Omi and Winant’s view of racial formations in the twenty-first century, considering race and racial distinction as socially constructed concepts formed through historical processes of racial categorization that have discursively associated different racial identities with distinct biological and cultural characteristics (Omi and Winant 2012). Anthony Marx also reasonably notes that the concept of racial formation challenges the extant assumption of race “as a pre-existing category” and that “race is not found, but ‘made’ and used (Marx 1996).” Considering race as a social construct, it is a concept of consciousness and perception that are linked to other identities of class, nationality, gender, as well as professionality, expertise, and competency. In turn, it is helpful to consider categorizations of race (be it whiteness or blackness) as results of a *becoming* through racial formations within social spaces rather than a biological force of nature.

In addition, Omi and Winant suggests that racial formation in the twenty-first century has become a covert process that is embedded within broader social structures under the scheme of colorblindness. Colorblindness “denies that race should inform perceptions, shape attitudes, or influence individual or collective action (Omi and Winant 2012).” It embodies a belief that “overt forms of racial discrimination are a thing of the past (Omi and Winant 2012).” Omi and Winant examines the case of Barack Obama’s administration—which served as a particularly racialized milestone and “proof” that the racial division has reconciled and hence racism has ceased to exist at the election of an African American president. As overt forms of racism became more and more unpopular, being perceived as racist became circumvented. Racism was avoided, neglected but not confronted nor really addressed, which allowed racism to be entrenched in the discursive, hidden within minds, perceptions, and seemingly race-neutral structures of hierarchies (Omi and Winant 2012). For humanitarianism, what Michael Barnett categorizes as its liberal transformation in the late twentieth century is arguably a similar kind of “milestone.” The exit of Cold War paved way for the emergence of an “international liberalism” that extolled “the virtues of autonomy, independence, and liberty” (Barnett 2011). In turn, any overt discourses that do not conform to liberal values, including oppressive racial stratifications intrinsically associated with imperiality and coloniality—would reasonably be frowned upon in a liberal humanitarian structure (Barnett 2011). In addition, I also view the contemporary humanitarian space similar to a structure of “empire,” in light of Barnett’s duly suggestion that although humanitarian governance legitimizes its actions through its purpose dedicated to emancipation and empowerment, it “does not depend on a process of deliberation, dialogue, or even consent (Barnett 2011).” The historical colonial dominance of the global South by European imperial states was nonetheless legitimized through the discourses of bringing civilization to the “savage” population, relieving them from their supposed backwardness. Humanitarian governance is a practice that is controlling in nature after all.

## The humanitarian workspace and practice

There is a growing body of literature recognizing the covert racial dynamics of the quotidian professional humanitarian structure that this paper intends to review. For example, Peter Redfield, through *The Unbearable Lightness of Expats*, uncovers how racialized inequalities and hierarchies in *Médecins Sans Frontières* (MSF) signified by the mobility of European expatriate staff that stand in sharp contrast to the immobility of the local, national staff working in the organization’s mission cites (Redfield 2012). Adia Benton offers a poignant argument for how race is closely intersected with other identities of nationality, citizenship, and class throughout the organizational processes of humanitarian practices (Benton 2016b). Patricia Ward has also duly highlighted that the category of ‘the local’ has been framed contrarily by organizations to best benefit their operations in different contexts that “subsequently reinforce and reproduce labor hierarchies articulated as local-expatriate, local-global, and even East versus West” (Ward 2020). Nonetheless, many literature examining contemporary aid, as well as the everyday lives of humanitarian professionals, reflect on the hierarchical divide along the lines of class exclusivity and foreign status prestige, although they not always accommodate the salience of race^2^. Benton reasonably emphasizes that while many researchers have addressed questions of race in professional humanitarian practice, “they have done so at the level of discourse, glossing racial hierarchies simply in terms of race masquerading as cultural difference rather than explicitly in terms of racialized practices and identification (Benton 2016b).”

Acknowledging this pronounced gap in the extant literature, the paper recognizes humanitarian professionals as those contractually employed to facilitate an aid program based on a set of eligibility criteria and regularly assessed for performance during their employment. This perspective allows for the identification for two important concepts for analysis: the professional humanitarian workspace and practice. The humanitarian workspace is highly transitional in nature, characterized by its *in-between*-ness, situated between the powerful and the powerless, the political and apolitical, as well as the reality of conflict and aspired peace. Lisa Smirl, in *Spaces of Aid*, suggests an “auxiliary space”—consisting of “cars, compounds and hotels”—that serves as a “rite of passage” for the practices of changemaking to be channeled from the “international” to the “local” (Smirl 2015). Within this auxiliary contains the overarching assumption that “space is malleable and static and that the production of new places can be disconnected from the techniques and processes used to produce it (Smirl 2015).” Through this lens, the examination of the everyday practices of humanitarian professionals as their own unique category of analysis becomes reasonable.

Emanuel Adler and Vincent Pouliot outlines five core characteristics of practices in international relations. They are (1) “a process of doing something” that is (2) patterned in a way that exhibits certain regularities over time and space. They are also (3) socially recognized in which their competence is “attributed in and through social relations.” Lastly, they (4) rest on the “intersubjective background knowledge” of practitioners (5) in both discursive and material worlds (Adler and Pouliot 2011). The characteristics of practice, particularly (3), (4) and (5), provide us with insights on the origins and nature of specific practices: they are normative, actionable products of what practitioners think they know based on their experiences of their social interactions and contextual understandings relevant to their identities. For Pouliot, practices in international relations are not necessarily always derived from “conscious deliberation or thoughtful reflection,” but instead, “the result of inarticulate, practical knowledge that makes what is to be done appear ‘self-evident’ or commonsensical (Pouliot 2008).” Therefore, the everyday practices of humanitarian professionals do not always result from rational choice-making of humanitarian professionals based on deliberate assessment of risk factors, as they can also be tacit, emanating from pre-existing assumptions that they hold. Humanitarianism is an ambiguous, broadly-encapsulating concept that can be interpreted differently—although it is often closely associated with humanitarian aid, as well as practices that reside in its peripheries, such as development, peacebuilding, and conflict intervention in crisis environments (Fast 2015). It is also in this discursive and moral ambiguity that contrasting values can co-exist, where the issue of race—and covert forms of racial stratification shrouded by the shadow of colorblindness—reside. Through this reasoning, tacit, assumptive practices of racialization can be distinguished from orchestrated, interpreted discourses of liberal humanitarian values.

The recognition of spatiality and practice allows for this paper to connect with broader literatures that focus on subjects and practices within the humanitarian “auxiliary space” that are tangentially connected to the discussion of racialized professional non-equivalences. For example, Eric James reflects on how the professionalization of the humanitarianism also enables the formulation of a hierarchy that is presided by those who can afford the acquisition of status symbols of institutional education and mobile, international working experience (James 2016). In addition, while Bandyopadhyay and Patil’s main focus of examination is on white female humanitarian volunteers, their findings can still signal a paternalism that paints underdevelopment and victimhood as indicators of incapability and unreliability in professional endeavors (Bandyopadhyay and Patil 2017).

The paper also features a number of responses from anonymized interviews with current and former humanitarian professionals. The list of interviewees includes humanitarian professionals of various levels of past experience, different fields of expertise, as well as distinct identities of gender, sexuality, and race, working in different country operations with different ranks within the organizations. A total of 18 respondents agreed to participate in remote video call interviews. The respondents were recruited through a combination of cold-contacting and “snowballing” methods: I requested respondents to refer me to other individuals within their own professional humanitarian networks if they felt comfortable doing so. A few participants were also my former colleagues in different aid programs. The participants were asked about how team meetings are organized, the compositions of their teams, the identity backgrounds of their peers in the office, as well as the working conditions and workload. They were also asked about how everyday work is organized within their organizations, as well as whether if they feel that their upward mobility and professional recognition is contingent upon their racial backgrounds^3^.

Taking a historical perspective, I will structure my examinations around local and BIPOC expatriate humanitarian professionals. I aim to demonstrate how expertise and competency is racialized within the humanitarian profession by reviewing relevant existing literature—particularly those that include ethnographic accounts on the everyday aspects of humanitarian work and life. I seek to (1) examine how the historical narrative of international “help” may perpetuate certain perceptions of (in)competency towards aid workers of different racial profiles. In addition, I will (2) review the impact of professionalization on status and expertise recognition within the humanitarian aid apparatus. The next part of the section begins with the personal account of Younus (pseudonym), my former colleague, as it embodies how racial perceptions are internalized among BIPOC humanitarian professionals. The conversation was very casual, and Younus’ responses were particularly significant because of this informality, meaning that it is unlikely that he deliberately prepared for and crafted his responses to my questions. In addition, discussions regarding racial inequalities within the international humanitarian community is significantly sensitive—if not formally avoided—so personal, off-the-record conversations can yield more genuine responses than formal interviews.

## The narrative of humanitarian help and devaluation of local expertise

In 2019, I was assigned by an international non-government organization (NGO) to its Ethiopia country office. During my posting, I worked extensively under the guidance of a local colleague, whom I will call by the pseudonym Younus, on a program that was soon to be concluded. After years of operation, the likelihood for the renewal of the program funding was low, which meant that Younus’ contract would soon terminate, and that he would have to look for other employment opportunities. To me, Younus possessed the qualifications for an expatriate program director position in Kenya at the same NGO that we were working for. Holding a graduate degree on nutrition and agriculture from Addis Ababa University, he had been in the aid sector for more than a decade. He had worked for multiple well-known international organizations, namely CARE, Save the Children, and World Food Programme, as well as UNHCR, managing and implementing a number of large-profile humanitarian and development aid programs. With a professional English proficiency and extensive knowledge of Kiswahili and Amharic, he was well-qualified for the expatriate position. Naturally, I suggested that he might be interested in exploring similar career options, to which he responded (Younus (pseudonym) 2019):

Oh no... they [the organization] would not hire someone like me. Maybe some guy from Europe, or America, with a good education and have seen the world. Someone like the deputy country director, he is an expert. He is more qualified.

The deputy country director—whom I will refer by the pseudonym Andrew—was a white, British, heterosexual man in his mid-thirties who oversaw all programs operated by our NGO in Ethiopia, and hence a supervisor of Younus. Although still a seasoned aid professional, it was Andrew’s first time in Ethiopia. He was years younger than Younus—who was approaching his late fifties—and acquired the deputy country director position 5 years after he first entered the humanitarian aid sector. In one way or another, Younus felt that his decade-long experience was not sufficient to qualify himself as an expert in comparison to Andrew, a white colleague “from Europe” with notably less field experience in Africa. His Ethiopian graduate degree seemed also to be not enough—as he indirectly implied that it was not a “good education” compared to one acquired in the West. Although he did not explicitly mention his race, Younus’ response exhibited a certain internalization of racial perceptions that devalued his work and experience as a black, African man.

Historically, the influx of international humanitarian organizations into the global South filled the institutional vacuums in newly independent and post-conflict developing states with Western professionals possessing expertise in various aspects of developmental governance. For years—and continuously in the present day—“African, Asian, and Latin American bureaucrats, practitioners, and even some activists come to be taught about their countries’ problems by people from the North (White 2002).” Accompanied with this dynamics was a perpetuation of the discourse that emphasized international humanitarian help—a dominating idiom implying that the international (and mainly the West) knows better in how to materialize good governance and sustainable development than the communities that have found themselves in crises that they did not necessarily cause.

The 2010 Haiti earthquake in Port-au-Prince is a sobering example. The international—and in particular American—humanitarian response was significant, although it came at the cost of constant and extensive international broadcasting of the repleted state that the earthquake has left for the local communities, particularly in the global West. Images of destroyed homes, hopeless locals, and seas of rubble, although efficient in garnering public attention and sympathy, also inherently signaled black Haitians as subjects pertinently dependent on Western charity (Balaji 2011). Haiti, already framed as a “lawless nation that could not function without assistance” before the earthquake, would become even more dysfunctional in face of a catastrophic natural disaster (Balaji 2011). As international humanitarian agencies—often reliant on Western governmental funding—legitimize their presence and need to intervene in communities of crises through mediated representations of fateful destitution in the global South, the development of a narrative that undermines the capability of locals to survive and recover from difficulties is almost inevitable (Mohanty 1991). However, this particular kind of narrative does not only influence how beneficiaries of aid and government administrations are perceived internationally. Local staff members working within the exact international humanitarian agencies that aspire to relieve the crises are also significantly affected, as they can be perceived as less competent, insufficiently professional, and more prone to corruption by their expatriate counterparts in their employing organizations (Benton 2016b).

Mark Schuller extensively examines this dimension under the Haitian context through the accounts of thirty Haitian NGO employees, stressing that the reason behind expatriate professionals’ condescension towards the competency of local staff members is ultimately a result of cultural imperialism within the humanitarian structure. Interviewees of Schuller’s research indicate that Western (and often white) expatriate professionals still reinforce ideas, interventions, and structures that are fundamentally foreign to Haitian localities. Many aid agencies responding to the Haitian earthquake promoted “particularly green foreigners above Haitians who, themselves, had intimate knowledge of the needs of their communities (Schuller 2016)^4^.”

Bandyopadhyay and Patil further emphasizes how the humanitarian narrative, in which “whiteness is associated with progress, power, and higher status,” and “those in the global South... have lower capacity for development,” overly celebrates the altruistic emotional morale of international, expatriate humanitarian workers at the cost of devaluing the expertise of their local counterparts (Bandyopadhyay and Patil 2017). Peter Redfield also accounts the observation of an Italian nurse working for Médecins Sans Frontières (MSF), reflecting upon her own status as a white, Western aid worker: “There’s a status of color in much of Africa, your authority and knowledge are rarely questioned when you are white (Redfield 2012).”

Humanitarian aid and development organizations are aware of the importance of their local staff members. After all, they know the roads, the community customs, the language (particularly in communities where neither French nor English are spoken), and the broader domestic political landscapes that organizations need to navigate through. Local knowledge, in turn, have become extensively highlighted by the operational narratives of most programs in crises environments (Harrison 2013). However, this narrative may perpetuate the existing local-expat hierarchical divide, as it further outlines the reason for local staff to continue serving in support-type positions, focusing on the coordination and implementation of program decisions made by expatriate leadership. In many ways, local professionals are valued for the field-level knowledge that they possess, but it does not necessarily translate to them being trusted with making administrative decisions based on their local knowledge to anchor the future trajectories of their organizations.

Humanitarian aid agencies cannot possibly operate without local knowledge and expertise—even though racialized perceptions of competency and expertise continue to undermine the presence of local humanitarian professionals. As agencies justify their presence in communities of crises through “repeated assertions of radicalized difference” between the international and the local, a covert discourse emerges to reinforce the idea that local expertise would only thrive with the leadership of expatriate thematic knowledge (Heron 2007). Professional categorizations “expatriate” and “local” are colorblind because they possess both separate and unequal statuses even when nowhere on paper—in contacts, regulations, policies—explicitly indicates as such. Yet at the same time, such separation and non-equivalence can be identified in structural and societal elements. The contemporary humanitarian discourse of international help overly emphasizes thematic, expatriate knowledge to specific, local expertise. As a result, local professionals receive comparatively less compensation, benefits, and protection and are almost perpetually bound to positions in the lower sections of the organizational hierarchy.

The growing advancement of communication technologies, particularly with the development of the Internet, has made information-sharing between field offices in the global South and agency headquarters situated in Western developed states much more affordable. Lisa, the director of a global forum researching aid worker security, recalls that many aid programs in the late 1990s and early 2000s significantly depended on local decision-making due to the high cost of international communication services (Interview by author, Lisa (pseudonym), 2020):

When I worked in an earlier aid program in Afghanistan—I think it was the late ninetieths—the only way for us to reach the UK headquarters of our organization was through phone dial. We had this one black satellite telephone which was so expensive to use for international calls, and we were advised to only turn to it when there is an absolute emergency, like if the country falls into a large-scale civil war over night. So naturally we were encouraged to relegate decision-making responsibilities to our national staff and find local solutions. But now as everything can be communicated via an email, the tasks of decision-making have since moved up in most organizations’ hierarchies.

The power and status hierarchy between expat and local humanitarian professionals is also addressed by Iman (pseudonym), a national program officer for the United Nations Office for the Coordination of Humanitarian Affairs (OCHA) in Syria. Expressing her concern regarding the decision-making process within her organization in light of the ongoing novel-coronavirus pandemic, she spoke in a hushed tone – even from her own home through a recent phone call with me (Interview by author, Iman (pseudonym), 2020):

All international organizations would develop a country plan to mitigate with new pandemic challenges. However, for us—at least in OCHA’s Damascus capital office—the country plan was developed in a closed-door meeting consisted of only expats. I was a bit shocked; I mean, at least they [the expat management] could have called some of the senior local staff to participate. After all, we have been around much longer than they have. But no, we were not even asked for comments or suggestions. What do they know about our country—where we are born and raised, in a conflict that we live through—that we do not know? Especially when most of them have only arrived several months ago?

It is not uncommon that expatriate professionals arrive in a new country program without familiarity of local geographical, social, and political dimensions. It is likely that most expats would have arrived in “the field” with no local knowledge at least once throughout their professional career. Some expatriate humanitarian professionals disclose this reality through their memoirs recounting their field experience. In *Emergency Sex*, Heidi Postlewait contended that she arrived in her posting as a secretary for the UN Mission in Cambodia while she “didn’t even know where Cambodia was (Cain et al. 2007).” Leanne Olson, through A Cruel Paradise, admitted that she “didn’t have a clue about Bosnia” when arriving for her Doctors Without Borders position (Olson 1999). In turn, the extent which the expatriate professionals understand local situations depend on how much they reference the knowledge of local staff in the short term and how well they assimilate to local life to perform their own observations in the long term.

This can be further illustrated by Ruth, a Ugandan doctor working for MSF, who disclosed to Peter Redfield that the organization’s expatriate administrative staff, and “particularly the French”, seemed to be notably incompetent, almost as if they were hired from “just off the streets in Paris (Redfield 2012).” Expatriate MSF staff in Uganda, during Ruth’s assignment, were also highly suspicious of local staff, often accusing them “when money had gone missing from a safe,” while arrogantly ignoring local advice on the designs and organizations of important building layouts (Redfield 2012). Silke Roth also noted that many local professionals would have to “train new international team members on a regular basis” while they themselves rarely have access to opportunities of being promoted to leadership positions that expatriate staff often occupy (Roth 2015). In fact, Ong and Combinido indicate that even though local professionals are equipped with the mental aptitude in navigating through new and unknown circumstances and living conditions, they still have “limited professional mobility within the global organization (Ong and Combinido 2018).”

The effects of local-expatriate staff categorizations also become particularly acute in the discussion on the agencies’ duty of care for their staff. While expatriate humanitarian professionals are often seen residing in walled compounds and following strict security procedures in crises environments, the majority of aid worker casualtiies often consisted of local professionals who do not necessarily receive equal amounts of protection. Yet at the same time, they “experience increased attack rates and fatality rates per capital relative to international staff, reflecting increased localization of aid in high-risk areas (Stoddard et al. 2019).” For example, Sally Mohsen, a local professional who had worked for Save the Children in Egypt during the Arab Spring, recounted how the agency’s local staff members were exposed to significant security threats due to the fact that they were not able to travel with the NGO-owned vehicles and asked to take public transportation instead. The logistics policy at the time was justified on the basis of funds limitations, but when being interviewed by Elettra Pauletto, Mohsen detailed that if “one of the international staff would travel they (the organization) would definitely assign a car (Pauletto 2018).” In fact, a majority of aid agency security policies are developed to address risks faced by expatriate professionals, as it is often assumed that local professionals are able to manage their own security (Stoddard et al. 2009). This overbearing expectation for local professionals, who themselves can be direct and indirect victims of the crisis environment, results in their disproportionally high security risk exposure.

Local humanitarian professionals, while taking on a significant bulk of operational tasks of aid programs, are also much less compensated compared to their international counterparts (Stoddard et al. 2006). In addition, they also receive less training, livelihood benefits, security provisions, and psycho-social support compared to their expatriate colleagues (Carr et al. 2010). Iman, in her accounts of working in OCHA Syria, has also disclosed to me that as a local staff, she is not provided with the opportunity to take advantage of the Rest and Relaxation (R&R) plan offered to her expatriate colleagues that allotted special vacation dates and subsidized air travel costs. In addition, besides being delegated noticeably similar (and often times higher) amounts of tasks than her expatriate colleagues, her salary is half of what expatriate staff with similar work experiences receive. For Iman, this material reality evidences the non-equivalence of status between expatriate (in her case, an all-white leadership team) and local humanitarian workers (Interview by author, Iman (pseudonym), 2020):

What many expatriate managements cannot fully comprehend is the fact that like all other local staff, I live the Syrian conflict twice: once as a humanitarian worker, and another as a Syrian national. Because of the instability, almost nowhere is safe, and yet everything is expensive. My family depend on me; if the conflict escalates, the expats get to go home to somewhere safe, but I have no other choice but to live through it.

Especially in an environment where waves of emergencies are seemingly endless, working overtime is common. In this context, unforeseen demands related to conflict and crisis response requiring immediate attention are easier to meet for those without daily family responsibilities. Expatriate professionals, who often arrive without family companions to serve in short-term postings, are more equipped to work overtime and complete more tasks in a fixed period of time should they choose to. However, this would frequently mean that expatriate management and leaderships who choose to work during “after hours” may expect similar working ethics as local staff, who are less compensated, less protected while having to shoulder their daily family responsibilities and navigate through everyday crisis challenges (Acker 2006).

Expatriate professionals, often arriving from a more developed part of the world (mostly North America or Europe), stand in a mobile, contingent contrast to the predictable, constant local professionals. They possess a notably privileged type of voluntary mobility; the migratory experiences of expatriate professionals for their respective aid works are rarely characterized by economic or political hardship. In turn, the much-celebrated, transcendent humanitarian worldview of selfless giving is more difficult for local professionals to take on, as they are both financially and socially disadvantaged to do so. For expatriate professionals, “the field” can be as specific as one community in crisis, or as ambiguous as any parts of the developing world, and that working in “the field” is an experience that can often be beneficial for the advancement of their career. For local professionals, on the other hand, “the field” is where they are born and raised, where their family reside, and where they live through the exact crises that they work to respond. As they struggle to conform to this humanitarian ideal, mitigating with the structural barriers within their profession and the social ties of their communities in crises, they may appear to be less-so committed, and hence less-so professional, than their expatriate counterparts. This in turn will affect their upward mobility within the organizational hierarchies within the professional aid sector.

Park, in his examination of the plight for professional recognition of volunteer health workers in the aftermath of the Ebola crisis in Sierra Leone, details how trained local nurses volunteered to work in Ebola treatment units operated by programs led by international aid agencies and the national government in hope to demonstrate their expertise and obtain permanent employment in the national health system. Instead, many were abruptly disengaged at the end of the epidemic without any emotional nor professional recognition (Park 2017). This phenomenon also occurs in the peacekeeping sector—as Coleman also stresses that many UN Volunteers, while often highly qualified individuals who perform “the same or similar types of functions as regular (international) staff” and “see their assignment as a possible path to a future United Nations staff career,” continue to receive formal warnings advising against such considerations (Coleman 2020).

The conflation of both fragmented human resource needs in face of unpredictable emergencies and inconsistent (and increasingly limited) funding available from donors, work contracts for local professionals are almost always short term (approximately 3 to 18 months) with uncertain likelihood for renewal (Korff et al. 2015). Local professionals, after dedicating significant resources and time to accumulate relevant certification and experience, nonetheless have to frequently apply for new assignments and be prepared for periods of unemployment and uncertainty in future prospects (Dickman et al. 2010). The increasingly pronounced career insecurity within the aid sector pressure local humanitarian professionals to conform to increasingly demanding work conditions without fair reciprocation from their employing international agencies in the forms of protection, recognition and compensation. Elisa Pascucci identifies this particular career instability as a form of “labor precarity” experienced by local aid workers that “reproduce a porous and contested ‘local vs. international divide” (Pascucci 2019). The differential labor divide between local and expat aid workers is founded upon deep-rooted structures of colonial and hierarchical stratification that raises “ethico-political concerns about the presumptions of abstract universality inherent to humanitarianism (Pascucci 2019).”

The most diligent few local professionals do manage to breakthrough into international postings or opportunities to pursue higher education in the developed world by excelling significantly at what they do. Their extraordinary achievements would be branded as “success stories” that justified the compromising work-compensation arrangements local staff often receive. The stories of the exceptional and lucky few serve as reminders for the majority of local staff who do not have international experience or higher education from recognized institutions in the global West to be content with their support and implementation roles, ultimately “reinforcing the idea that a job in a global agency is a ‘blessing’ (Ong and Combinido 2018).” Yet at the same time, most expatriate professionals from countries in the global West, like Leanne Olson and Heidi Postlewait, though undoubtedly experts with thematic competency, still manage to retain significant global mobility in the humanitarian sector without necessarily having specific local expertise.

Without what Autesserre terms as the “knowledge hierarchy” that places thematic competency over local expertise, the idea of deploying expatriate professionals to a country that they have never lived in or even heard of would seem absurd. In reality, one NGO recruiter explained to Autesserre that familiarity with local dynamics is “neither a prerequisite nor a necessity” for expatriate professionals in crisis intervention and relief at large (Autesserre 2014). In her most recent book, *The Frontlines of Peace*, Autesserre further elaborates this issue in the specific context of peacebuilding, noting that often times, “as far as promotions go, most peacebuilding agencies reward the number of missions completed in different countries rather than the amount of time spent in a particular area.” In addition, she also observes that many “interveners discredit foreigners who stay too long in a specific place... as having ‘gone native’—implying that they are too immersed in the local culture, and too close to host populations, to effectively carry out their mission (Autesserre 2021).” This mindset has long encouraged the unwarranted discrediting of local expertise and increasing preference for top-down, thematic knowledge—preferred for their generalizability and simplicity in crisis environments that are, in contrast, unique and complex. This exemplifies Barnett’s observation that “devastation invites re-formers to imagine new arrangements that can peel away the causes of suffering and create the spiritual and material foundations for a better world (Barnett 2011).” In turn, international humanitarianism is not necessarily aiming to rebuild communities in crises as they were, but instead to paint new and supposedly improved pictures on the blank slates created by devastation.

The local aid worker imagery are perceived to possess contrasting and conflating qualities in different contexts and occasions (Ward 2020). At the international level, in front of public stakeholders, donors, and everyone external to the humanitarian workspace, local aid workers are highly valuable and essential professionals that make humanitarian operations possible. Within the organizational hierarchies, local aid workers are relegated to the roles of support, assistance, and service. There is hence a hybridity within the discourse: the “local” is both the victims of war, the embodiment of underdevelopment, yet the most knowledgeable in facilitating change “on the ground.” Ilan Kapoor well-nuances this hybridity through reasoning with Homi K. Bhabha, suggesting that it highlights the conflating, unstable nature of colonial and imperial discourses that possess polarizing yet co-existing enunciations—hence, “in the very practice of domination the language of the master becomes the hybrid (Kapoor 2002; Bhabha 1994).” Although local aid workers’ existence is recognized to be necessary, their inclusion in the higher stratifications of international humanitarian leadership is nonetheless constantly resisted. In many ways, Barnett is reasonable to suggest that humanitarian governance today “has a chummy relationship with the very empires that it supposedly resists (Barnett 2011).”

Therefore, their competency and knowledge are not the sole factors that determine the ability for local professionals to participate in organization-wide decision-making processes. Their “localness,” characterized by their black, brown, and non-white skins, can be the ultimate determinants to local experts’ professionalism being recognized and respected, as their racial profiles have long been formed in strong association with certain origins of under-development. While expatriate professionals *may* be BIPOC, local professionals are *always* BIPOC: the expat-local divide is intrinsically founded upon historically formulated perceptions of race and color, blinded by the exact discourses of “help” and “liberation” extolled by contemporary liberal humanitarianism.

## The special plight for legitimacy and recognition of BIPOC expatriate professionals

There is no race-aggregated information on the identity profiles of aid workers currently employed within the international humanitarian sector^5^. However, we do know that at the beginning of post-Cold War liberal humanitarianism, Western, white personnel occupied almost all leadership, higher consultancy and advisory positions for humanitarian aid operations in crises contexts (Kothari 2006; Redfield 2012). The end of the Cold War is a historical milestone for the institutionalization and non-politicization of global humanitarian governance (Barnett 2011). Professionalization of aid, in turn, can also be considered as a product of the post-Cold War institutionalization of humanitarian aid. General Roméo Dallaire, the French-Canadian force commander for the United Nations Assistance Mission in Rwanda (UNAMIR), is a notable representation of the imagery of an international humanitarian leadership. Another example would be the “cowboy doctor” imagery forged by earlier MSF operations—a rugged, cigarette-smoking French man, troubled by the conflict he has witnessed as he roamed through communities in crises, providing independent, emergency treatment for the sick in underdeveloped countries (Bortolotti 2010). Predominantly Western, white (mostly male and heterosexual) humanitarians set a precedent of what expatriate expert leaderships looked like for members of host communities, including local humanitarian workers. In addition, David, Schroeder and Fernandez suggest that racism, especially those developed through historical, colonial discourses, may perpetuate racial prejudice to the point where subjects of oppression internalize racist perceptions (David et al. 2019). Consequently, the existing racial perceptions of host communities internalized through their colonial pasts further consolidates the linkage between whiteness and humanitarian expertise leadership.

Omi and Winant’s framework of racial formation is particularly fitting in this historical perspective: within the humanitarian profession, whiteness has been racially formulated in the leadership positions of rebuilding communities in crises. In turn, whenever BIPOC and non-Western expatriate humanitarian professionals arrive, they challenge the status quo racial profile of whiteness assumed for the positions they serve in. Although not completely uniform, white professionals are nonetheless “often treated as if they have a higher status irrespective of their position (Crewe and Fernando 2006).” Uma Kothari provides a concerning account of her personal experience working as a BIPOC development consultant, where her local counterparts had been “visibly disappointed when they realized that their expatriate consultant was not white (Kothari 2006).” She further notes that local staff often equate the appointment of a white consultant to represent a higher status to their work, and that receiving a consultant of other racial profiles would mean that their organization is devalued (Kothari 2006). Amare Tegbaru also reflects on this issue based on his 10-year experience working as a BIPOC expatriate rural development advisor for FAO in Thailand, during which he was treated very passively by his local counterparts in comparison to his white expatriate colleagues. As a Swedish national, Tegbaru did not deny his Ethiopian origins and was not hesitant to disclose that his partner is also not white. In turn, he did not possess the visual characteristics—nor connections to—the stereotypical profile of an expatriate advisor to his local counterparts (Tegbaru 2020). He was less attended to in his office, and at one point his workstation was moved from an area reserved for expatriate staff to a noisy corridor occupied by mostly local-level support staff, namely drivers, storekeepers, and cleaners. Although he eventually gained the trust of his local counterparts through his work, he was always described based on his Western, Swedish background, while his African origin was noticeably downplayed. “They were not happy with me highlighting my African background while in their company in public; they preferred that I use my Swedishness and field of expertise as the most appropriate self-description (Tegbaru 2020).” In his case, Tegbaru’s professional competency was almost a compensation for his blackness that enabled him to be perceived as a professional of equal capacity to other white expatriates.

Kothari and Tegbaru’s personal accounts exhibit a concerning reality. White expatriate professionals receive initial leadership status recognition; BIPOC expatriate professionals can find themselves pressured to prove their Western connections and “whiteness” through demonstrating their expertise in order to garner the same recognition from local peers and political elites. A British, Asian development worker, in the words of Crewe and Fernando, would be described by her West African peer to “have a white brain (Crewe and Fernando 2006).” At the same time, a Ugandan team leader would have to dress in “smart” Western attires and speak commandingly in English instead of the local language to assert the authority she needs to perform her professional functions (Redfield 2012). There is hence what Ngugi wa Thiong’o terms as the “colonization of the mind’—whereas in the professional humanitarian space, competency, expertise, as well as higher moral values are associated to whiteness by people with past experiences of colonial oppression (wa Thiong’o 1986). In turn, when a BIPOC expatriate humanitarian professional demonstrates their expertise, they are not just competent—they are competent *like white expatriates*.

At the same time, they can also be subjects of racial prejudices from their white peers. Adia Benton outlines this dimension by presenting her own observations in the field, when her NGO received its first African country director after a series of mainly young, white male predecessors. Upon his initial arrival, the competency of the African country director was extensively questioned by white expatriate staff members in the organization, who held longstanding assumptions that personnel “from poor countries did not have the aptitude to lead complex institutions, manage diverse groups of people, and advance organizational missions (Benton 2016b).” For Clarke, Perreard and Connors, a core determinant of the professionalization that have taken place in the humanitarian space is the emergence of the discourse recognizing that communities in crises deserved the best care possible (Clarke et al. 2019). This would explain the existence of standards or requirements for certifications and degree diplomas for humanitarian work qualifications—which are nonetheless means that agencies have taken on to mitigate with operational risks and retain at least some forms of identifiability and legitimacy. This in turn creates an elitism that, Eric James suggests, “can negatively affect workers who have not had the same pay or education, or gained a similar level of disaster experiences and group memberships... people who do not possess the ‘appropriate’ status symbols may be seen as ‘not professional’ (James 2016).” In addition, the growing number of educational programs focused on humanitarian aid and peacekeeping in growing numbers of universities in the global West is a notable characterization of such professionalization (Walker et al. 2010).

However, who is able to access such type of institutionalized training? There are very realistic, resource-oriented concerns in training and certifications: those who can pay for entry fees and practice courses put them at a considerable advantage to those who cannot. Similarly, degree programs in humanitarianism or peacekeeping, as well as internships that are often unpaid, come at a cost that many cannot afford. Moss, Uluğ, and Açar are reasonable to emphasize that in conflict contexts, “very often academic training does a poor job preparing us for field research (Moss et al. 2019).” Yet, this reality can extend to any professional field-level postings in humanitarian, peacekeeping, and development sectors. In contexts of complex emergency and conflict where situations are frequently shifting and new developments emerge on a regular basis, the room for aid workers to apply previously acquired, “cookie-cutter” solutions and best practices is significantly limited. Consequently, their work often involves the facilitation of relationships, networks, and connections among other pertinent actors within the humanitarian space. Such “people relations” skills can rarely be systematically acquired in an institution setting removed from the actual conflict context. It is fair to say that the pursuit of self-legitimization by humanitarian organizations through the professionalization of humanitarian work have promoted an elitist transformation of the practice as accessible only for those who can afford to become “competent.” Paradoxically, the profession of humanitarian work that aims to assist those who are marginalized also prevents them from joining the practice—or as Hugo Slim notes, through professionalization, humanitarian work has become equally inclusive and exclusive (Slim 2015).

Assumed lack of professional experience and educational background are, therefore, not necessarily the fundamental reasons behind the covert disapproval subjected to BIPOC expatriate professionals by their white counterparts, especially for those coming from or visibly resemble the people of the global South. Roth explained how a European professional of Asian descent was ignored when attending an expat party in an Asian country until she was “introduced as being from Europe and working for a UN agency,” as she was initially assumed to be a local (Roth 2015). Although white professionals are automatically included in the expat bubble, BIPOC expatriates need to present connections to the West or other statuses known to be unattainable by locals first (Heron 2007). To be part of the expat bubble, they need to first “code-switch” their narratives, appearance, and expressions to what are normally considered appropriate behaviors for expatriate professionals. Essentially, the ticket to the expat bubble is not necessarily demonstrated expertise, but a status difference from locals. They may need to ride in SUV cars with special status license plates, have a local driver, and subscribe to a certain lifestyle afforded to them by their expatriate salary to symbolically compensate for their color (Tegbaru 2020). Ultimately, in order to join the club and be recognized by their expatriate peers, BIPOC expatriate professionals often need to signal that while they look like locals or peoples from other parts of the global South, the resemblance stops there. In most scenarios, BIPOC expatriate humanitarian professionals find themselves delicately positioned between (1) having to demonstrate their professional competency and (2) needing to signal their expatriate status in order to garner the recognition they need to function in their leadership positions. The former often requires them to work closely and learn from local staff serving in support and implementation roles, while the latter would likely require them to distinguish themselves from local staff. This “double-bind” is a challenge often unique to BIPOC expatriate humanitarian professionals, requiring them to find a delicate balance between signaling the expatriate status and duly fulfilling their professional responsibilities.

## Concluding reflections

Every aspect of the paper concerning expertise and professional recognition can be discussed in a “color-less” way in writing. Competency, experience, and qualification can be framed as identity-neutral, merit-based concepts that define an aid worker’s professionalism. However, what it takes to be recognized as professionals and experts is undeniably subjective and perceptive. This paper, in the reflection of what exactly does it take for an aid worker to be professionally recognized, sought to uncover the covert barriers in the road to recognition that have long been filtering BIPOC aid workers from their professional upward mobilities.

The contemporary humanitarian apparatus has always subscribed to the narratives of diversity, inclusion, and equality that rendered overtly racist remarks in the workplace inappropriate. The problem, however, is that it often obfuscated all discussions regarding race, even those that sought to reasonably address legitimate non-equivalences between expatriate and local professionals within organizations founded upon racial prejudices at the structural level. Within this dynamic, it is visible that an acute form of *colorblindness* has long legitimized the perception that “any hints of race consciousness are tainted by racism; hence, most anti-racist gesture, policy or practice is to simply ignore race (Omi and Winant 2012).”

Humanitarianism arguably aspires to embody altruistic ideals of care and philanthropy. However, this aspiration is at the constant tension against the persistent influence of humanitarianism’s imperial and colonial histories, as well as its institutionalized and professionalized contemporary reality. The aim of the research is not to completely disregard the aspirations and potential of humanitarian work. However, it nonetheless challenges the broad-based assumption that the working experiences of humanitarian professionals are unproblematic. Aid agencies and humanitarian professionals do not necessarily form unified fronts in face of the crises that they aspire to relieve. Not everyone necessarily joins the humanitarian professional sector solely for an altruistic cause, holding the same values of care and inclusivity that are often considered synonymous to humanitarianism.

The expat-local divide exemplifies a deeper racial prejudice within the internal structures of international aid agencies that is not purely contingent on a professional’s foreign or local status. The practice of inward socializing among white, Western professionals within the exclusivity of the expat bubble highlights how status and recognition for expertise is very much centered around one’s whiteness and the mistrust towards the aptitude of BIPOC professionals. In fact, I isolated race specifically for the purpose of this discussion, believing that it is notably under-examined in the context of contemporary global humanitarian governance, where the “crude modernization view of the global South nevertheless lurks within the ‘discursive bricolage’ of development (White 2002).” Examining humanitarian aid as a career industry can also allow us to apply existing theories on organizational structures, professionality, human resource management, and workplace dynamics. Through these approaches, we can better understand how existing practices, narratives, and habits of humanitarian professionals are not only based on what they know—but also what they perceive and assume.

Race is an identity factor of a broader intersectionality also consisting of gender, class, nationality, ethnicity, and sexuality that all play respective roles as indicators of inequality within the professional humanitarian space. I have carefully debated on isolating the concept of race in my examination of the structure of contemporary aid apparatus, which is already fragile, under extensive international scrutiny while it possesses at least some semblance of altruism. Barnett, in his analyses of the historical evolutions of humanitarianism, rebuffs both its romanticization and condemnation. Instead, he treats humanitarianism as “a morally complicated creature, a flawed hero defined by the passions, politics and power of its times even as it tries to rise above them (Barnett 2011).” Indeed, humanitarian aid is often associated with concepts of care, altruism, and unconditional giving, but it nonetheless finds itself with colonial, imperial underpinnings of rule and control. In the specific case of race, the humanitarian narratives should not cover the professional hierarchies and status stratifications that are contingent upon the racial profiles of aid workers.

It is important to recognize that covert racial dimensions within organizational structure is something that needs to be seen in-person more than read through texts. Adia Benton effectively combines both images and textual descriptions in her analysis of the racialized non-equivalences within the humanitarian profession (Benton 2016a). This research recognizes Benton’s note on the importance of imagery—but it hopes to demonstrate that color can nonetheless be brought into writing. However, the only way for written literature to account for racial dimensions is to subscribe to explicit indications of racial profiles (such as black, white, brown, Latino or Asian). Not openly addressing race in the discussion regarding the professional non-equivalences between expatriate and local professionals is, after all, an active choice made by particular organizations, professionals and researchers.

Recalling the 2003 Assembly for the MSF in the USA, Redfield accounts that a speaker urged to distinct “culturally unaware, eyes-closed behavior,” and “intentional domination” from expatriate professionals to stress that the term neocolonialist may be a strong term to reference the behaviors or attitudes of certain expatriate professionals in the field (Redfield 2012). Yet, being culturally unaware and eyes-closed is nonetheless significantly concerning as a characteristic for an expatriate staff in contemporary humanitarian governance. BIPOC humanitarian professionals, both local and expatriate, see and feel the racialization of their expertise and professionalism through daily lives—such as when they newly arrive in their country office, during lunch breaks, when meeting with local political counterparts for the first time, and through daily conversations with other constituents of aid. Although expertise and competency are not directly visible, it is often associated with visible characteristics. In today’s humanitarian space, one’s whiteness can be a very covert yet common prerequisite for professional recognition, all the while “ideas about black inferiority precede professional encounters (Benton 2016b).” Barnett duly suggests that “humanitarianism is a creature of the very world it aspires to civilize (Barnett 2011).” In an avid attempt to move forward towards a world that we aspire for, we have perhaps left the reality behind, unwilling to acknowledge that the impact of colonial, racial prejudice persists in the post-colonial world. Altruistic values and inclusive narratives do not automatically erase structural problems for a humanitarian space that has evolved into a professional industry. Humanitarian, peacekeeping, and development organizations, as well as individual professionals working within these sectors, would be in a much better position to address the needs of communities in crises if they are aware of their own prejudices. For organizations that operate within uncertain crises contexts, knowing the problem is ultimately much better than being oblivious to it. Within the contemporary humanitarian space lives a certain *colorblindness*, ignoring that racism is still very much alive and, more dangerously, covertly woven within its social fabrics, implicitly disadvantaging BIPOC professionals working in a sector that aspires to embody values of altruism.

## Data Availability

Records and transcripts of the interviews performed for the research will not be shared in accordance to the consent to participate agreement between the interviewees and the author. The topics discussed during the interviews are of potentially sensitive nature, and may negatively impact the interviewees if directly published.

## Footnotes

1 For this paper, “global South” is understood as areas of the world hosting states with shared colonial pasts and experiencing considerable underdevelopment as a result of social and political instability. This paper also takes on the perspective of Dependency Theory, seeing the root causes of global South underdevelopment and instability to be the dominance and dependency relations between poorer, weaker states and richer, more powerful states that are mostly situated in the global West.

2 On the expat-local divide, as well as how such divide exhibits a class and nationality-based status elitism in humanitarian and development aid organizations, see Kennedy (2019), Voorst (2019) and Harrison (2013)

3 The interviews were facilitated as part of an Master of Arts thesis at McGill University, titled “High Aspirations and Low Expectations: Coping and Living in the Everyday Politics of Humanitarian Fieldwork.” The thesis explores how the identities of humanitarian professionals impact their perceptions of risks—which in turn influence their everyday practices in crises environments. The issue regarding the racialization of expertise and professional non-equivalence is one of three areas of examination that composed of the empirical analysis of the thesis.

4 Schuller (2016), Roth (2015), Autesserre (2014), as well as Kothari (2006) all present similar dimensions of non- equivalence of compensation between local and expat professionals in crisis intervention and humanitarian aid. For example, (Schuller 2016) mentions the housing allowance for an expatriate NGO country director can be as high as 8,000 US dollars a month.

5 For example, reports of the UN Secretary General on the staff demographics of the UN Secretariat do not feature a race component in its data.
